# Nighttime Bracing or Exercise in Moderate-Grade Adolescent Idiopathic Scoliosis: A Randomized Clinical Trial

**DOI:** 10.1001/jamanetworkopen.2023.52492

**Published:** 2024-01-29

**Authors:** Anastasios Charalampidis, Elias Diarbakerli, Marlene Dufvenberg, Kourosh Jalalpour, Acke Ohlin, Anna Aspberg Ahl, Hans Möller, Allan Abbott, Paul Gerdhem

**Affiliations:** 1 Department of Clinical Science, Intervention and Technology (CLINTEC), Karolinska Institutet, Stockholm, Sweden; 2 Department of Reconstructive Orthopaedics, Karolinska University Hospital, Stockholm, Sweden; 3 Unit of Physiotherapy, Department of Health, Medicine and Caring Sciences, Linköping University, Linköping, Sweden; 4 Clinical and Molecular Osteoporosis Unit, Department of Clinical Sciences, Malmö, Lund University, Lund, Sweden; 5 Department of Orthopaedics, Skåne University Hospital, Malmö, Sweden.; 6 Department of Orthopaedics, Ryhov Hospital, Jönköping, Sweden; 7 Stockholm Center for Spine Surgery, Stockholm, Sweden; 8 Department of Orthopaedics, Linköping University Hospital, Linköping, Sweden; 9 Department of Orthopedics and Hand Surgery, Uppsala University Hospital, Uppsala, Sweden; 10 Department of Surgical Sciences, Uppsala University, Uppsala, Sweden

## Abstract

**Importance:**

Moderate-grade adolescent idiopathic scoliosis (AIS) may be treated with full-time bracing. For patients who reject full-time bracing, the effects of alternative, conservative interventions are unknown.

**Objective:**

To determine whether self-mediated physical activity combined with either nighttime bracing (NB) or scoliosis-specific exercise (SSE) is superior to a control of physical activity alone (PA) in preventing Cobb angle progression in moderate-grade AIS.

**Design, Setting, and Participants:**

The Conservative Treatment for Adolescent Idiopathic Scoliosis (CONTRAIS) randomized clinical trial was conducted from January 10, 2013, through October 23, 2018, in 6 public hospitals across Sweden. Male and female children and adolescents aged 9 to 17 years with an AIS primary curve Cobb angle of 25° to 40°, apex T7 or caudal, and skeletal immaturity based on estimated remaining growth of at least 1 year were included in the study. Dates of analysis were from October 25, 2021, to January 28, 2023.

**Interventions:**

Interventions included self-mediated physical activity in combination with either NB or SSE or PA (control). Patients with treatment failure were given the option to transition to a full-time brace until skeletal maturity.

**Main Outcomes and Measures:**

The primary outcome was curve progression of 6° or less (treatment success) or curve progression of more than 6° (treatment failure) seen on 2 consecutive posteroanterior standing radiographs compared with the inclusion radiograph before skeletal maturity. A secondary outcome of curve progression was the number of patients undergoing surgery up until 2 years after the primary outcome.

**Results:**

The CONTRAIS study included 135 patients (45 in each of the 3 groups) with a mean (SD) age of 12.7 (1.4) years; 111 (82%) were female. Treatment success was seen in 34 of 45 patients (76%) in the NB group and in 24 of 45 patients (53%) in the PA group (odds ratio [OR], 2.7; 95% CI, 1.1-6.6). The number needed to treat to prevent curve progression with NB was 4.5 (95% CI, 2.4-33.5). Treatment success occurred in 26 of 45 patients (58%) in the SSE group (OR for SE vs PA, 1.2; 95% CI, 0.5-2.8). Up to 2 years after the primary outcome time point, 9 patients in each of the 3 groups underwent surgery.

**Conclusions and Relevance:**

In this randomized clinical trial, treatment with NB prevented curve progression of more than 6° to a significantly higher extent than did PA, while SSE did not; in addition, allowing transition to full-time bracing after treatment failure resulted in similar surgical frequencies independent of initial treatment. These results suggest that NB may be an effective alternative intervention in patients rejecting full-time bracing.

**Trial Registration:**

ClinicalTrials.gov Identifier: NCT01761305

## Key Points

**Question:**

Is self-mediated physical activity combined with either nighttime bracing or scoliosis-specific exercise superior to self-mediated physical activity alone for preventing Cobb angle progression in moderate-grade adolescent idiopathic scoliosis?

**Findings:**

In this randomized clinical trial that included 135 patients, aged 9 to 17 years, who were skeletally immature with moderate-grade adolescent idiopathic scoliosis, nighttime bracing combined with self-mediated physical activity prevented curve progression of more than 6° to a significantly greater extent than did self-mediated physical activity alone, while scoliosis-specific exercises did not.

**Meaning:**

Results of this study suggest that nighttime bracing is an effective alternative intervention in patients rejecting full-time bracing.

## Teaser

The randomized clinical trial assesses whether self-mediated physical activity combined with either nighttime bracing or scoliosis-specific exercise is superior to self-mediated physical activity alone for preventing Cobb angle progression in moderate-grade adolescent idiopathic scoliosis.

## Introduction

Adolescent idiopathic scoliosis (AIS) is a 3-dimensional (3D) structural deformity of the spinal column of unclear etiology, affecting otherwise healthy children and adolescents (hereinafter youths) during their growth spurt. The prevalence of AIS is approximately 3%,^1^ and approximately 10% of those with AIS develop a more aggressive deformity requiring treatment.^2^

Previous studies have suggested that full-time bracing is effective in the treatment of patients with moderate-grade AIS.^3,4,5^ Among patients not accepting full-time bracing, nighttime bracing (NB) has been proposed as an alternative treatment. Nighttime bracing could counteract compliance issues and the impact on quality of life observed in patients receiving full-time bracing.^2,6,7^ To our knowledge, there have been no randomized clinical trials investigating NB vs a control group.

Scoliosis-specific exercise (SSE) primarily involves training in 3D postural self-correction strategies of the scoliosis curvature, stabilization of corrected postures, and their integration into activities of daily living including physical exercise.^8^ It is unknown whether SSE may prevent progression of moderate AIS.^9^ The aim of this trial was to determine whether self-mediated physical activity combined with either NB or SSE was superior to a control of physical activity alone (PA) in preventing Cobb angle progression in moderate-grade AIS.

## Methods

### Study Design

The Conservative Treatment for Adolescent Idiopathic Scoliosis (CONTRAIS) randomized clinical trial was conducted in 6 public hospitals (Karolinska University Hospital, Linköping University Hospital, Ryhov Hospital Jönköping, Eskilstuna Hospital, Västmanlands Hospital in Västerås, and Sundsvall Hospital) across Sweden.^10^ Institutional review board approval was obtained by the Regional Ethical Board in Stockholm (see trial protocol in Supplement 1). Written informed consent was obtained by patients and parents. The study conformed to the Consolidated Standards of Reporting Trials (CONSORT) reporting guideline for nonpharmacological clinical trials.^11^ Male and female youths with previously untreated moderate-grade AIS were included in the study if they had a primary curve Cobb angle of 25° to 40° with apex T7 or caudal, were aged 9 to 17 years, and were skeletally immature with estimated remaining growth for at least 1 year and not more than 1 year after menarche for female youths. Patients with all types of scoliosis curves could be included as long as they fulfilled the aforementioned criteria. Patients were excluded if their scoliosis was an etiology other than idiopathic, as determined by clinical information, clinical examination, or medical imaging, or were unable to understand Swedish. Before participation, patients eligible for the study had their radiographic evaluations assessed at the principal investigation center by 2 experienced investigators (P.G. and H.M. or A.C.). Randomization was done in a 1:1:1 ratio. The randomization sequence was prepared a priori by an independent statistician. Opaque envelopes containing an intervention group number were prepared by the same independent statistician. The original randomization list was kept by the independent statistician and was unknown to the research personnel. The randomization envelopes were opened in the presence of at least 2 persons. Blinding of patients and therapists for treatment was not possible in this study. Patients who were eligible but not willing to participate in the study were offered standard care with a corrective thoracolumbar sacral orthosis and were instructed to wear it for at least 20 hours each day. Inclusion of patients in the study began January 10, 2013, and terminated October 23, 2018.

### Study Interventions

Detailed information^12^ about the interventions is provided in the appendices of the published protocol^10^ and 6-month interim patient-reported outcomes.^13^ Patients in the NB group received a custom-designed, hypercorrective Boston scoliosis night brace (Camp Scandinavia AB).^14^ The brace was prescribed to be worn for 8 hours during the night. An orthotist was available for brace adjustment when needed. Introduction and adjustment of the brace were conducted in an outpatient or inpatient setting by the same orthotist at each site throughout the study period. A senior orthotist (Mats Hoffsten, BSc, a member of the CONTRAIS Study Group) approved the radiological in-brace correction for all patients treated with a brace at all sites.

Patients in the SSE group were educated using a motor-learning approach to perform active self-correction of their scoliotic posture in 3D planes, muscular stabilization of the corrected posture, and integration in a home exercise program 30 minutes daily. The intervention was initially guided by an experienced physiotherapist during 3 individual sessions of 90 minutes once every month during the first 3 months and additional guided sessions were conducted if needed. This was to ensure the patient’s capability to perform and self-manage a daily home exercise program with support of parents.

Patients in the PA group were encouraged to perform physical activity for at least 60 minutes daily, according to World Health Organization recommendations,^15^ for the entirety of the study. The NB and SSE groups were encouraged to fulfill the same quota of physical activity.

To facilitate adherence to the study interventions, education and support provided to patients and parents were aimed at optimizing the capability, opportunity, and motivation behavior model.^16^ Reinforcement and progression of the assigned intervention were performed at each 6-month follow-up.

### Data Collection

Radiographic, anthropometric, angle of trunk of rotation,^17^ and patient-reported data were collected at baseline and at each 6-month follow-up. Assessment of radiographs was conducted by the treating health care practitioner at each 6-month follow-up. The primary outcome was curve progression of 6° or less by skeletal maturity (treatment success) or curve progression of more than 6° seen on 2 consecutive posteroanterior standing radiographs compared with the inclusion radiograph (treatment failure). Radiographic measurements were conducted through radiographic images in the Digital Imaging and Communications in Medicine image format using the picture archiving and communication system, version 23.1 clinical imaging tool (Sectra PACS). For the patients in the NB group, the brace was not worn the night before the radiograph was conducted. All radiographs of cases with suspected treatment failure were assessed by 2 experienced investigators (P.G. and H.M. or A.C.) unaware of the treatment prior to any decision on treatment failure. Patients who experienced treatment failure were given the option to transition to a corrective thoracolumbar sacral orthosis, worn at least 20 hours per day, instead of the assigned treatment, until confirmation of skeletal maturity.

Skeletal maturity was defined as less than 1 cm of growth of body height in 6 months. Body height was measured by a wall-mounted standard stadiometer at each center.

Health care practitioners reported the grade to which the patient adhered to the treatment plan (reported patient adherence), ranging from best (very high: 1 point) to worst (not at all: 4 points), using information from a patient diary and after dialogue with the patients and families. Patient-reported treatment adherence, motivation to carry out treatment, and capability to perform treatment were also collected using a similar scale. These data were collected at each 6-month follow-up.^13^

Patients and practitioners could respond to an open-ended question regarding adverse events at each 6-month follow-up. All patients were followed up until 2 years after the time point of the primary outcome. Transition to a full-time brace and occurrence of surgical intervention for scoliosis were noted.

Other secondary outcomes (not reported here) included generic and disease-specific patient-reported outcome measures such as the child-friendly European Quality of Life-5 Dimensions instrument,^18^ the Scoliosis Research Society-22r instrument,^19^ the International Physical Activity Questionnaire–Short Form,^20^ a modified Spinal Appearance Questionnaire,^21^ and the Cobb angle^22^ of the major curve at the end of the study.

After the end of the study, independent assessment of all radiographs was made by 2 experienced physicians (K.J. and A.O.), who were blinded to treatment allocation, using Surgimap spine software, version 2.3.2.1 (Nemaris Inc), into which all radiographs had been exported. To ensure measurement agreement between the 2 blinded assessors, we conducted an interrater reliability test; a 2-way mixed-effects model was used to calculate the intraclass correlation coefficient (ICC) with 95% CI on measurements of the Cobb angle of the major curve at inclusion. The ICC ranges between 0 and 1, with values below 0.5 indicating poor reliability; between 0.5 and 0.75, moderate reliability; between 0.75 and 0.9, good reliability; and above 0.9, excellent reliability.^23^ The same model was then used to determine measurement agreement between health care practitioners and the 2 blinded assessors at 2 time points: inclusion and the end of the study. The ICC for the 2 blinded physicians who conducted the Cobb angle measurements was 0.86 (95% CI, 0.79-0.89) for the major curve at inclusion. The ICC for the mean value of the Cobb angle measurements performed by the 2 blinded physicians and the Cobb angle measurements by the health care physicians was 0.91 (95% CI, 0.87-0.93) for the major curve at inclusion and 0.96 (95% CI, 0.94-0.97) for the major curve at the end of the study.

### Statistical Analysis

Dates of analysis were from October 25, 2021, to January 28, 2023. The statistical analysis plan and power calculation are available with the a priori protocol.^10^ In our primary analysis, which was an intention-to-treat analysis, all participants, regardless of noncompliance, loss to follow-up, or dropout, remained in the analysis of the group to which they were randomized. A sensitivity analysis was performed comparing the ITT data against per-protocol data exclusively from patients who complied with the study protocol.

Baseline categorical parameters were compared by a χ^2^ test. Continuous and discrete parameters were measured using parametric or nonparametric tests for group comparisons.

Odds ratios (ORs) and 95% CIs for treatment success (≤6° Cobb angle progression by maturity) in the NB group and the SSE group, compared with the PA group, were calculated. Kaplan-Meier survival and log-rank test analyses were used to display the probability of curve survival of 6° or less over time for each group (treatment success).

A dichotomous variable was created based on whether health care practitioners reported patient adherence as a very high grade and high grade or a low grade and not at all at each 6-month follow-up until the primary outcome was reached. Similar dichotomization was performed on postrandomization covariates such as patient-reported treatment adherence, motivation to carry out treatment, and capability to perform treatment. This was applied in an additional sensitivity analysis with complier average causal effect (CACE) weighting to provide estimates of the treatment effect among compliant participants without breaking randomization.^24^ The CACE and per-protocol analyses were adjusted for age, Risser grade (a skeletal maturity assessment based on the ossification of the iliac apophysis; grades range from 0-5, with higher grades indicating higher maturity),^25^ Cobb angle of the major curve at inclusion, and sex.

All statistical analyses of the primary outcome were conducted by 2 independent statisticians (Per Näsman, PhD, and Henrik Hedevik, MSc, members of the CONTRAIS Study Group; see Supplement 3), who were blinded to intervention and assigned to each patient participating in the study. SPSS Statistics, version 28 (IBM Corp); SAS, version 15.2 (SAS Institute Inc); and RStudio package in R, version 4.2.0 (R Project for Statistical Computing) were used for the analyses. A 2-sided *P* < .05 was considered statistically significant.

## Results

### Patient Characteristics

**Figure 1.**
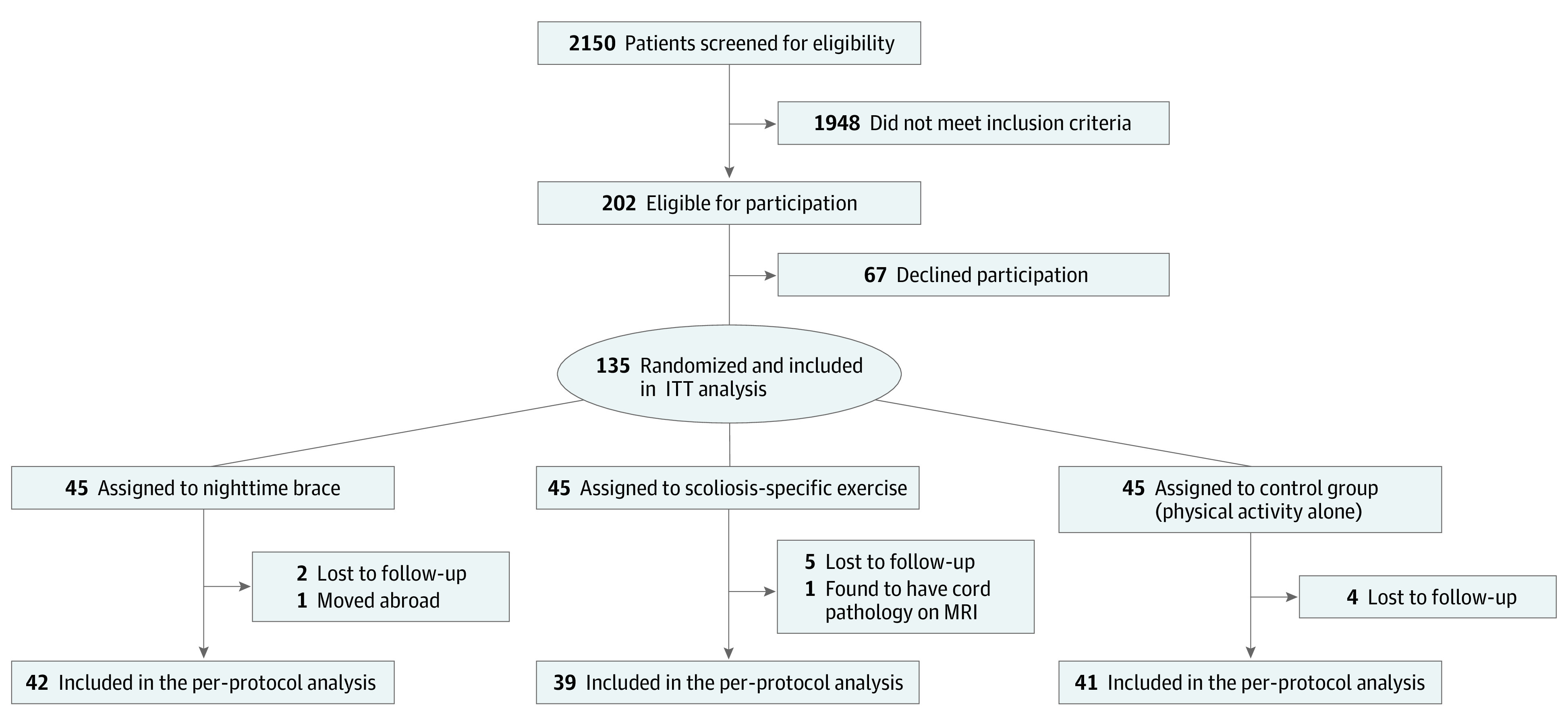
CONSORT Diagram of Trial Participation From January 10, 2013, to October 23, 2018, patients were screened in 6 study centers (Karolinska University Hospital, Linköping University Hospital, Ryhov Hospital Jönköping, Eskilstuna Hospital, Västmanlands Hospital in Västerås, and Sundsvall Hospital) across Sweden. CONSORT indicates Consolidated Standards of Reporting Trials; ITT, intention to treat; MRI, magnetic resonance imaging.

From January 10, 2013, through October 23, 2018, 202 patients met the inclusion criteria, and 135 participated in the study (Figure 1). Baseline characteristics are presented in Table 1. The mean (SD) age for the entire cohort was 12.7 (1.4) years, 111 (82%) were female, and 24 (18%) were male. During the trial, 13 participants dropped out of the study (Figure 1). The per-protocol analysis included 122 patients: 42 in the NB group, 39 in the SSE group, and 41 in the PA group.

**Table 1.** Baseline Characteristics for the Entire Cohort and Intention-to-Treat Population

Characteristic	Overall sample (N = 135)	NB group (n = 45)	SSE group (n = 45)	PA group (control; n = 45)
Age, mean (SD), y	12.7 (1.4)	12.7 (1.4)	12.6 (1.4)	12.6 (1.5)
Sex, No. (%)				
Female	111 (82)	39 (87)	33 (73)	39 (87)
Male	24 (18)	6 (13)	12 (27)	6 (13)
Height, mean (SD), cm	158 (10)	157 (9)	158 (10)	159 (10)
Weight, mean (SD), kg	46.0 (9.2)	44.8 (9.3)	45.7 (9.0)	47.3 (9.4)
BMI, mean (SD)	18.3 (2.6)	18.0 (2.7)	18.2 (2.4)	18.6 (2.7)
Angle of trunk rotation, mean (SD), °	11 (3)	12 (3)	11 (3)	11 (3)
Risser grade, No. (%)^a^				
0	68 (53)	21 (50)	22 (52)	25 (57)
1	14 (11)	5 (12)	4 (10)	5 (11)
2	14 (11)	5 (12)	3 (7)	6 (14)
3	23 (18)	9 (21)	8 (19)	6 (14)
4	9 (7)	2 (5)	5 (12)	2 (4)
5	0			
Cobb angle of the major curve, mean (SD), °^b^	31 (4)	32 (4)	31 (4)	31 (4)
Location of the major curve, No. (%)				
Thoracic	92 (68)	31 (69)	31 (68)	30 (67)
Thoracolumbar	27 (20)	9 (20)	7 (16)	11 (24)
Lumbar	16 (12)	5 (11)	7 (16)	4 (9)

Abbreviations: BMI, body mass index (calculated as weight in kilograms divided by height in meters squared); NB, nighttime bracing; PA, physical activity alone; SSE, scoliosis-specific exercise.

^a^ The Risser grade is a staging system of bone maturity based on the ossification of the iliac apophysis, ranging from 0 to 5, with higher grades indicating greater maturity. Data were missing for the Risser grade in 7 patients.

^b^ Analysis of variance with the Dunnett correction test did not show any significant difference in the mean Cobb angle of the major curve between the NB group or the SSE and PA groups.

### Primary Outcome

#### ITT Analysis

The success rate in the NB group was 34 of 45 patients (76%) and 24 of 45 patients (53%) in the PA group (OR, 2.7; 95% CI, 1.1-6.6) (Table 2). The number needed to treat to prevent 1 case of curve progression with the nighttime brace was 4.5 (95% CI, 2.4-33.5), and the relative risk reduction was 48% (95% CI, 4%-71%). The mean (SD) time in the study was 22.8 (12.9) months in the NB group and 16.2 (10.5) months in the PA group (*P* = .01). The success rate in the SSE group was 26 of 45 patients (58%; OR for SSE vs PA, 1.2 [95% CI, 0.5-2.8]) (Table 2). The mean (SD) time in the study was 16.1 (10.6) months in the SSE group vs the PA group (*P* = .99).

**Table 2.** Outcomes in the NB, SSE, and Control Groups

Outcome^a^	Group, No. (%)			*P* value^b^	
	NB	SSE	PA (control)	NB vs PA	SSE vs PA
Intention-to-treat analysis (n = 135)					
Success	34 (76)	26 (58)	24 (53)	.03	.67
Failure	11 (24)	19 (42)	21 (47)		
Per-protocol analysis (n = 122)					
Success	31 (74)	20 (51)	20 (49)	.02	.82
Failure	11 (26)	19 (49)	21 (51)		

Abbreviations: NB, nighttime bracing; PA, physical activity alone; SSE, scoliosis-specific exercise.

^a^ Success was defined as 6° or less of curve progression before skeletal maturity and failure as more than 6° of curve progression before skeletal maturity.

^b^ Pearson χ^2^ test was used.

#### Per-Protocol Analysis

The per-protocol analyses yielded similar results as the ITT analyses (Table 2). The success rate in the NB group was 31 of 42 patients (74%) and 20 of 41 patients (49%) in the PA group (OR, 2.9; 95% CI, 1.2-7.4). The mean (SD) time in the study was 24.1 (12.3) months in the NB group compared with 17.2 (10) months in the PA group (*P* = .007). The success rate in the SSE group was 20 of 39 patients (51%) (OR for SSE vs PA, 1.1; 95% CI, 0.4-2.6). The mean (SD) time in the study was 17.8 (9.8) months in the SSE group compared with the PA group (*P* = .95).

#### Kaplan-Meier and CACE Analyses

**Figure 2.**
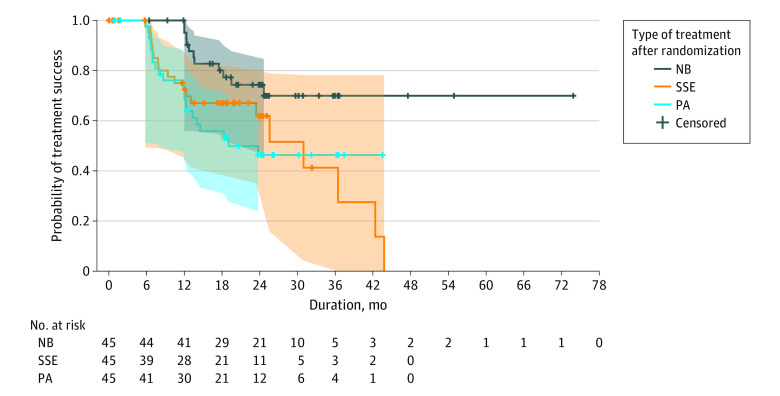
Kaplan-Meier Survival Analysis The cumulative proportion of patients with treatment success (≤6° of Cobb progression) in the 3 groups. Survival distributions for the nighttime bracing (NB) group and the control group with physical activity alone (PA) were significantly different: log-rank test, 7.3 (*P* = .007). The survival distributions for the scoliosis-specific exercise (SSE) group and the PA group were not significantly different: log-rank test, 0.2 (*P* = .65). Hash marks indicate censored data; shaded areas, 95% CIs.

The results of the Kaplan-Meier analysis and the log-rank test are shown in Figure 2. The pooled logistic regression ITT analysis adjusted for baseline covariates showed that had everyone been assigned to the NB group, the hazard of progression would be 0.17 (95% CI, 0.07-0.43; *P* < .001) times the hazard of progression had everyone been assigned to the PA group over the 6-month follow-up periods until the primary outcome was reached. When this ITT analysis was weighted for treatment adherence (CACE), the hazard of progression would be 0.16 (95% CI, 0.05-0.52; *P* = .002), and similarly per-protocol would be 0.14 (95% CI, 0.04-0.44; *P* < .001). The pooled logistic regression ITT analysis adjusted for baseline covariates showed that had everyone been assigned to the SSE group, the hazard of progression would be 0.91 (95% CI, 0.43-1.93; *P* = .80) times the hazard of progression had everyone been assigned to the PA group over the 6-month follow-up periods until the primary outcome was reached. When this ITT analysis was weighted for treatment adherence (CACE), the hazard of progression would be 0.58 (95% CI, 0.20-1.63; *P* = .30), and similarly per protocol would be 0.57 (95% CI, 0.21-1.58; *P* = .28) (eTables 1 and 2 in Supplement 2).

#### Adverse Events

In the complete cohort, 19 adverse events were reported in 15 patients from treatment start until the primary outcome was reached, of which 7 adverse events were reported in 7 patients during the first 6 months. In the NB group, 16 adverse events (trunk pressure and skin problems [n = 10], sleeping problems [n = 2], emotional problems [n = 1], shoulder and neck pain [n = 2], and unspecified [n = 1]) were reported in 12 patients. In the SSE group, 3 adverse events were reported (pain during treatment [n = 1], muscle strain [n = 1], and low back pain [n = 1]) in 3 patients, while no adverse events were reported in the PA group.

#### Follow-Up After the Primary Outcome

Allowing transition to full-time bracing after treatment failure resulted in similar surgical frequencies independent of initial treatment. In the immediate period after curve progression (treatment failure), 6 of the patients in the NB group transitioned to full-time bracing, while 4 were treated with surgery; 11 patients in the SSE group transitioned to full-time bracing, while 6 were treated with surgery; and 14 patients in the PA group transitioned to full-time bracing, while 6 were treated with surgery. At 2 years after the primary outcome time point, 9 patients in each group had been treated with surgery.

## Discussion

In the CONTRAIS randomized clinical trial of 135 male and female youths with moderate-grade AIS, NB produced a greater success rate in preventing progression of the scoliotic curve compared with a control group. Of note, there was no statistically significant difference in success rate for SSE compared with the PA group.

While longer hours of full-time brace wear have been shown to be superior to observation in terms of preventing curve progression,^5^ many patients experience resistance to adhering to full-time dosage or reject using a full-time brace altogether.^2,4,6,26^ Evidence on the effectiveness of NB is scarce in the literature, provided mainly by heterogeneous studies retrospective in nature and not controlled. In the studies, success rates with NB were between 52% and 89%,^27,28,29,30,31^ similar to the success rate of 76% seen in the present trial, while the success rate of observation was only 50%.^7^ The findings of the present study have direct clinical implications, as we provided evidence on the effectiveness of NB to reduce the risk of curve progression in patients with moderate-grade AIS. To our knowledge, this was the first fully randomized clinical trial that investigated the effectiveness of bracing for the treatment of moderate-grade AIS. Previous randomized clinical trials either failed to recruit patients and ended randomization early^32,33,34^ or started as a randomized clinical trial and ended as a randomized clinical trial combined with a preference cohort.^5^

In the present trial, SSE did not result in a higher success rate in preventing scoliosis progression compared with the PA group. Scoliosis-specific exercise has been suggested to reduce curve progression, but its efficacy is still unclear.^35^ In mild curves, studies have reported positive outcomes, supporting that SSE may prevent scoliosis progression.^9,36^ In moderate curves, 1 prospective randomized clinical trial compared SSE with full-time bracing alone and found a greater reduction in the Cobb angle in the brace group over a 12-month follow-up.^37^ To our knowledge, there have been no other prospective studies on the effectiveness of SSE compared with a control group with longer follow-up until skeletal maturity and 2 years beyond maturity.^38^

### Limitations

This study has some limitations. The block randomization process used in this trial may be criticized, as the allocation of participants may have been predictable, but investigators were blinded to block size. Compliance with the treatment plan did not include objective measures. While overestimated self-reported adherence can be expected,^39,40^ both patient and health care practitioner reports were used, and there was still a clear advantage of NB in CACE analyses, indicating that even in cases with suboptimal compliance, bracing was more effective than other treatment modalities. Our primary outcome guaranteed patient safety, allowing transition to full-time bracing as evidence supporting a beneficial effect of a full-time brace has emerged since the study start.^5^ The option to transition to a full-time brace after treatment failure may also be the reason that our 2-year posttreatment analysis did not show any difference in the surgical rates among the 3 groups. The content of the SSE intervention may also be criticized, since we synthesized a broad exercise treatment protocol without any preference for a specific treatment regime. However, the synthesis of this protocol was based on the best available evidence at the time we designed the study with the aim to be applicable in an outpatient setting.

## Conclusions

In the CONTRAIS randomized clinical trial involving 135 youths with moderate-grade AIS, NB demonstrated a higher success rate compared with a control group in the prevention of curve progression, while SSE did not. These findings suggest that NB may be an effective alternative intervention among patients rejecting full-time bracing.

## Notes

Supplement 1.
Trial Protocol

Supplement 2. **eTable 1.** Causal Survival Analysis for Nighttime Bracing Compared With Self-Mediated Physical Activity **eTable 2.** Causal Survival Analysis for Scoliosis-Specific Exercise Compared With Self-Mediated Physical Activity

Supplement 3. **Nonauthor Collaborators.** CONTRAIS Study Group members

Supplement 4.
Data Sharing Statement
