# Supplementary material for: Nighttime Bracing or Exercise in Moderate-Grade Adolescent Idiopathic Scoliosis: A Randomized Clinical Trial
Source: JAMA Netw Open. 2024 Jan 29;7(1):e2352492. doi: 10.1001/jamanetworkopen.2023.52492 (PMC10825714; doi:10.1001/jamanetworkopen.2023.52492)
Supplement: Supplement 1. — Trial Protocol [file jamanetwopen-e2352492-s001.pdf]

Purpose and aims

The purpose of this project is to fill knowledge gaps identified in the literature regarding the conservative treatment of idiopathic scoliosis. Recent systematic literature reviews on physical exercise and bracing in the treatment of idiopathic scoliosis have provided some low quality evidence for their efficacy in preventing the progress of scoliosis and reducing surgery rates. There is however a clear lack of randomised controlled trials comparing the effectiveness of these interventions.

From a health care delivery and patient related perspective, knowledge of the treatments effect in terms of cost effectiveness, quality of life, psychological wellbeing and biophysical changes is lacking. Furthermore, it is unknown which patient related, situational, or environmental characteristics moderate treatment effects and which processes mediate treatment effects. This specific knowledge could help in developing more personalised and efficient health care intervention delivery which may influence treatment outcomes more positively.

The primary aim of this research proposal is to improve evidence for the costs and effects of conservative treatments for idiopathic scoliosis which may help prevent the progression of scoliosis and the need for surgical interventions. Secondary aims include improving knowledge of factors moderating and mediating treatment effect in terms of patients quality of life, psychological wellbeing as well as biophysical factors.

Survey of the field

Idiopathic scoliosis affects around 3% of children and adolescents. Idiopathic scoliosis occurs in otherwise healthy children and is often diagnosed at the time of the pubertal growth spurt. About one tenth gets a more aggressive variant leading to a more severe deformity of the spine and thorax. By early adulthood, the majority of patients with scoliosis suffer from back pain and if the curve progresses to be very large, even pulmonary dysfunction and psychological distress can occur [1]. Treatment in severe cases is brace or surgery.

In its mild form, idiopathic scoliosis is common and is not treated. In Sweden, at least 200,000 children are screened yearly by school nurses and physicians. Minimum requirement is school screening in grades 4 (age 10-11) and in grade 7 or 8 (ages 13-14) [2]. The aim is to find the children with moderate scoliosis. These are treated with a brace to prevent progression to severe scoliosis. It can be estimated that about 3,000 Swedish children are referred to orthopaedic surgeons for further investigations, often including x-rays of the spine. About 300-500 children start brace treatment yearly.

More severe curves are treated with spinal fusion surgery. About 300-400 are surgically treated for idiopathic scoliosis yearly in Sweden. Surgical complications are few but if they occur, may be devastating, such as paraplegia [3]. The spinal curve is corrected and fused with limitation in mobility as a result (Figure 1).

Figure 1. (A) Radiograph of moderate idiopathic scoliosis in adolescent girl. (B) Boston brace (usage at least 20 hours per day). (C) Night time brace (usage eight hours per day). (D) Radiograph in night time brace showing large correction of the curve. (E) Severe idiopathic scoliosis. (F) Result after corrective deformity surgery. The surgery took approximately 5 hours.

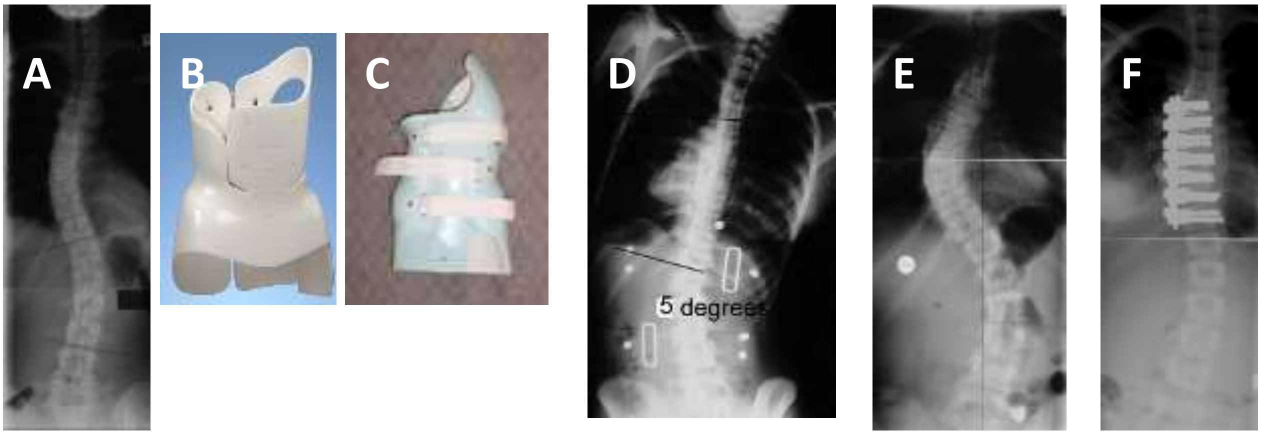

Several theories propose that during the adolescent period of skeletal growth, bone deformation may occur in the event of vertebral body weakness or an imbalance of muscle forces as well as joint flexibility. A recent review of literature concerning the association of low bone mineral density and idiopathic scoliosis reported an osteoporosis prevalence of 20-38 percent [4]. Physical activity is a requirement for normal growth and development during childhood and adolescents. It is well documented that physical exercise is associated with improvements in not only muscle strength, aerobic fitness and motor development but also bone density which may help decrease the risk of osteopenic related bone deformation [5, 6].

A recent report from the Swedish National Institute of Public Health (SNIPH) showed that only 45% of 11, 13 and 15 year olds are physically active 60 minutes per day, 5-7 days a week. An additional 30 percent were physically active 3-4 days a week and 25% 0-2 days a week [7]. Literature even suggests that patient's with idiopathic scoliosis treated conservatively and those treated with surgery have lower participation in sports activities than age matched controls [8]. Guidelines from the SNIPH and the World Health Organisation (WHO) recommend a minimum of 60 minutes of physical activity every day. Both moderate and strenuous activity should be included and can be divided up into several shorter sessions during the day. Activities should be enjoyable and as varied as possible to provide aerobic fitness, muscular strength, flexibility, speed, shorter reaction times and coordination [9, 10]. Moderate-intensity physical activity is referred to as working hard enough to raise your heart rate and break a sweat, yet still being able to carry on a conversation.

Interventions using a social cognitive theory based approach including health educational and motivational approaches to improve adolescent's habitual physical activity level have been recommended based on previous literature [6]. Such interventions include changes on both a school based level and even health care based level. The Swedish government have even implemented legislation for changes in school physical education programs focused on offering students the opportunity for daily physical activity throughout the hole school day as a complement to physical education classes.

In the case of idiopathic scoliosis, it is possible that newly diagnosed patients with reinforcement

from parents may automatically restrain from physical activity in concern for it contributing to scoliosis progression. Research has however shown that systematic exercising is not associated with

the development of idiopathic scoliosis [11]. Orthopedic teams involved in longitudinal screening of scoliosis progression therefore need to play a main role in educating and motivating patients with the support of parents and teachers to maintain recommended physical activity levels to minimize the possible contributing effects of inactivity.

In general, conservative treatments such as physical exercise or bracing, aim to prevent and possible reverse progression of scoliosis curves. Published guidelines for the conservative treatment of scoliosis recommend that conservative treatments for idiopathic scoliosis be implemented when curve magnitudes of 25-45° are apparent [12]. Surgical intervention for idiopathic scoliosis is generally first considered when curve magnitudes reach >50° [13].

A recent systematic literature review investigating the effectiveness of physical exercise in the treatment of idiopathic scoliosis reported the results of one randomised controlled trial, nine prospective cohort controlled trials, eight prospective observational cohort studies and two retrospective cohort studies [14]. Together, the studies provide a limited evidence base which suggests the efficacy of physical exercise in reducing the scoliosis progression rate and/or reducing the curve size compared to observation. It is however unclear if there is a difference in the effect of physical exercise alone or if physical exercise needs to have a postural corrective approach. Similarly, a recent literature review investigating the effectiveness of brace treatment of idiopathic scoliosis reported the results of two prospective cohort controlled trials and 18 longitudinal case control studies [15]. Together, the existing studies provide a limited evidence base for the efficacy of bracing preventing the progression of scoliosis without reduced quality of life.

Two randomized trials comparing brace treatment with observation are currently being conducted, one in Canada (http://www.controlled trials.com/ISRCTN81 733841/scoliosis) and one in the US (http://www.srs.org/professionals/positions/?id=62). Another trial from Holland has been terminated due to recruitment difficulties [16].

Both of the on-going randomized trials use a Boston brace like orthosis worn during day and night as the active treatment (Figure 1). The patients in the control arm are observed only. The Boston brace is made of hard plastic and stretches from under the arms to the pelvis. It is custom made and corrects the scoliotic curvature of the spine when worn. The psychological impact when using the brace 20-23 hours per day should not be underestimated. In one study, 27% of the brace treated patients reported that the treatment had a major negative effect on their lives [17], and our clinical impression is that this is one of the reasons to the poor compliance that is often seen. Recently, preliminary data from Gothenburg suggested that approximately eight hours of night-time bracing with an over-corrective brace was as effective as bracing during 23 hours per day [18].

Night-time bracing is attractive since you wear the brace a limited amount of time (Figure 1). The brace does not restrict activities during daytime. Our clinical impression is that the psychological concern for a teenager is much less when compared to brace treatment day and night-time, which increases the possibility of good compliance. There have been no controlled studies on night time bracing versus observation only. Several uncontrolled trials have been published, indicating an immediate corrective effect on the scoliosis by the brace [19] (Figure 1).

Only one low quality study has compared bracing with physical exercise, showing no statistical differences in the reduction or progression of scoliosis curves between the groups. To draw valid conclusions about the effectiveness of postural specific physical exercise and brace therapeutic interventions compared to a self mediated activity observational group, a randomised controlled

trial research design is needed to compare the interventions.

The orthopaedic clinic at the Karolinska University Hospital is responsible for scoliosis treatment in the Stockholm County. The yearly number of referrals concerning children with suspected scoliosis from school health care, orthopaedics, or primary health care is around 600.

Project description

Research questions

Are there significant differences in outcomes for idiopathic scoliosis patients receiving postural corrective physical activity or brace treatments, and what differences exist compared to a group that performs non-specific physical activity?

Research design

Newly referred patients to the Karolinska University Hospital orthopaedic clinic receive an orthopedic consultation including x-ray assessment of the spine. The magnitude of the curve in the frontal plane is assessed by measuring the Cobb angle [20]. Patients between 8-17 years of age with a primary curve of 25-40° with no prior treatment and skeletal immaturity assessed by the level of ossification on wrist x-rays as well as pelvic x-ray. Patients with estimated remaining growth of at least one year will be eligible for the study.

Patients will be excluded from the study if the pathogenesis of the scoliosis is not idiopathic but due to a neuromuscular, neurological, congenital malformation or trauma related comorbidity.

After assessment, patients fulfilling the inclusion and exclusion criteria will be asked to give written and signed consent for participation in an open book randomised controlled trial comparing three forms of health care management which are current practice in different clinics internationally. On recruitment to the study, baseline measures will be taken and random concealed block allocation using sealed opaque envelopes will be used to form the intervention groups. We estimate that all participants have been recruited in approximately 3 years. Patients will be reassessed every 6 months.

The end point failure of treatment is defined as an increase of the Cobb angle of at least 6 degrees on two consecutive x-rays, when compared to the x-ray performed at time of inclusion. Based on figures from previous literature, a failure rate of 45% in the observational self-mediated activity group and a 15% failure rate in the brace and postural training group is hypothesised. Given a significance level of 5%, a power of 80% and consideration for dropout of up to 20%, an estimated 45 patients are required in each of the intervention groups. Patients choosing not to participate in the study due to the randomisation process will be observed clinically at the same time points as the participants in the intervention study. For this observational group, self mediated activity will not be encouraged, so the natural course of these patients will be followed. Patients recruited to the brace group but not adhering to the intervention protocol will cross-over to the observational group recommended to continue self-mediated physical activity. Patients who have Cobb angle progress >6 degrees and ultimately reaching the end point failure of treatment will not remain in the study but will be offered Boston brace treatment. In the event that a Cobb angle surpasses 50 degrees, patients will be offered surgical treatment.

Figure 1. *Flow chart of the randomised controlled study*

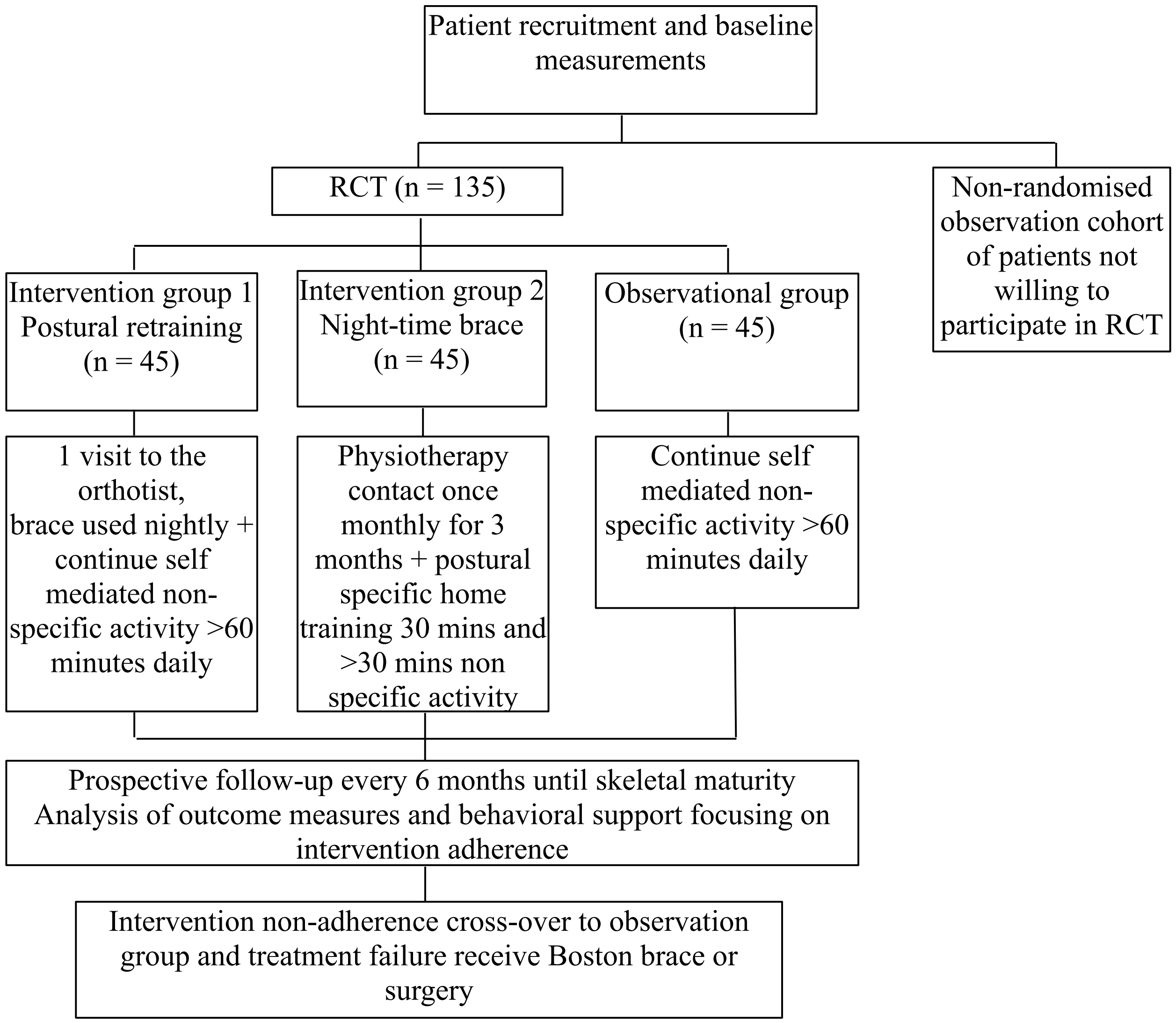

Interventions

1. Postural corrective physical activity:
The first phase of the intervention will be delivered in 3 x 1 hour sessions, once per month during the first 3 months. Goals at the neuromotor and biomechanical levels are directed towards postural control and spinal stability, while the goals at the bodily and psychological levels are directed towards aerobic functioning and development of a positive body image. The following steps are used:

Session 1

- Postural rehabilitation: Training awareness of body posture and postural deficits by using visual (mirror) and tactile (contact in the various postures) and verbal (therapist) feedback.
- Active self-correction on the 3 spatial planes:
  - Training of the awareness of curve apex translation towards concavity on the frontal plane. For example, in the case of a double-curve scoliosis, teaching how to execute thoracic curve horizontal translation and then lumbar curve horizontal translation.
  - Training awareness of correction on the sagittal plane to ensure thoracic kyphosis and lumbar lordosis within normal ranges. With the patient leaning with their back against a

wall, the patients train pelvis anteversion and a kyphotisation movement at the thoracic level. When the patient becomes aware of the movement, it is then done without feedback from the leaning against the wall.
  - By combining the previous movements in the frontal and sagittal planes, cross-sectional derotation then occurs in the third plane.
- Training muscular stabilisation and endurance in corrective postures: Muscle endurance strengthening aims at developing paravertebral, abdominal, lower limb and scapulo-humeral girdle muscles through isometric contractions in lying, sitting and standing positions. It uses loads that are one-third to two-thirds of maximal load in active self-correction. Patients are asked to execute an active self-correction movement and to hold it for the entire duration of isometric contraction of the chosen muscles.
- Training muscular stabilisation and endurance in corrective postures during closed kinetic chain weight bearing functional movements
- Self-mediated home postural training program to be performed daily until session 2.

<u>Session 2</u>
Development of balance reactions: Training muscular stabilisation and endurance in corrective postures during open and closed kinetic chain functional movements on unstable planes, developed with growing difficulties. Self-mediated home postural training program to be performed daily until session 3.

<u>Session 3</u>
Neuromotor integration: Progressively developing the ability to apply active auto-correction through integration in everyday behaviours such as during walking, running, lifting, oculo-manual tasks and sport specific tasks. During this conclusive phase of treatment, ergonomic education is also given. The postural specific self-mediated home training program is to be performed with moderate intensity at least for 30 minutes daily, for the entirety of the study. The patients are even recommended to continue with other non-specific self-mediated physical activities previously performed prior to the study >30 minutes daily to fulfill the general recommended quota of >60 minutes moderate intensity physical activity per day. A training diary will be implemented to follow and motivate the patient's training behaviour.

The second part of the intervention is the follow-up assessment and a cognitive behavioural therapy approach to the reinforcement of the postural training and other self mediates activities. The patients training diary will provide a basis for motivational discussion. This will be performed in conjunction with clinical reassessments every 6 months.

2. Brace treatment
An over-corrective brace will be specifically designed to the patient’s individual scoliosis type and will be prescribed for night time use. A cognitive behavioral therapy approach to reinforcement of brace use will be performed in conjunction with reassessment every 6 months. Patients are encouraged to also continue with non-specific self-mediated physical activities of moderate intensity at least 60 minutes daily, for the entirety of the study. A training diary will be implemented to follow and motivate the patient's training behaviour.

3. Non-specific self-mediated physical activity
Patients are encouraged to also continue with non-specific self-mediated physical activities of moderate intensity at least 60 minutes daily, for the entirety of the study. A cognitive behavioral therapy approach to reinforcement of physical activity will be performed in conjunction with reassessment every 6 months. A training diary will be implemented to follow and motivate the patient's training behaviour.

Study 1: Measurements
The following measures will be used to assess treatment outcome and to assess possible moderation and mediation of treatment effects.

Background data: The following data will be collected at the first visit after inclusion in the study:
- Patient demographics such as age, gender, weight, height, health status and medications

Primary outcome measures recorded at baseline and every 6 months for the entirety of the study:
- Cobbs angle: The Cobb angle is formed by the inclination of the upper end plate of the upper end vertebra and the inclination of the lower end plate of the lower end vertebra measured on posterior to anterior view x-ray pictures. The end point failure of treatment was defined as an increase of the Cobb angle of at least 6 degrees from the time of the first x-ray on two consecutive x-rays.

Secondary outcome measures recorded at baseline and every 6 months for the entirety of the study :
- SRS-22 quality of life questionnaire
- EQ5D /-Y/ quality of life questionnaire
- International Physical Activity Questionnaire (IPAQ) short form.
- Surgical rates

Treatment effect modifiers:
The following biophysical and psychosocial measures will be performed at baseline and every 6 months after the start of the intervention:
- Biophysical measures
  - 3D Surface topography picture sequence
    - Static postural indices in 3 spatial planes
    - Spinal proprioception for sense of repositioning
    - Test of trunk stiffness to the application and removal of a load during a progressive isoinertial lifting evaluation (PILE)
  - Isometric muscle endurance (Sorensen test, Shirado test)
  - Test of balance: Static, clinical balance is tested with sharpened Romberg's position with eyes closed and the non-dominant foot in front of the dominant foot. Dynamic, clinical balance is tested walking in a figure-of-eight.
  - Physical exertion test. Use of measures of Borg's RPE (rating of perceived exertion)
  - Spirometry

- Psychosocial variables
  - Mental health subscale of the SRS-22
  - SAQ – Scoliosis appearance questionnaire

At each 6 month follow-up additional questions regarding protocol fulfilment, patient and health care worker satisfaction and adverse affects will be asked:
- Adherence to the intervention protocol.
- Use of external health care services.
- Use of medicines (analgesics).
- Report of adverse events and symptoms.

Time Plan

Under the duration of 3 years it is estimated that 135 patients can be recruited to form the 3 patient groups. The following represents a timeline for the project (Figure 2).

Figure 2. *Research program timeline (numbers refer to study numbers mentioned above).*

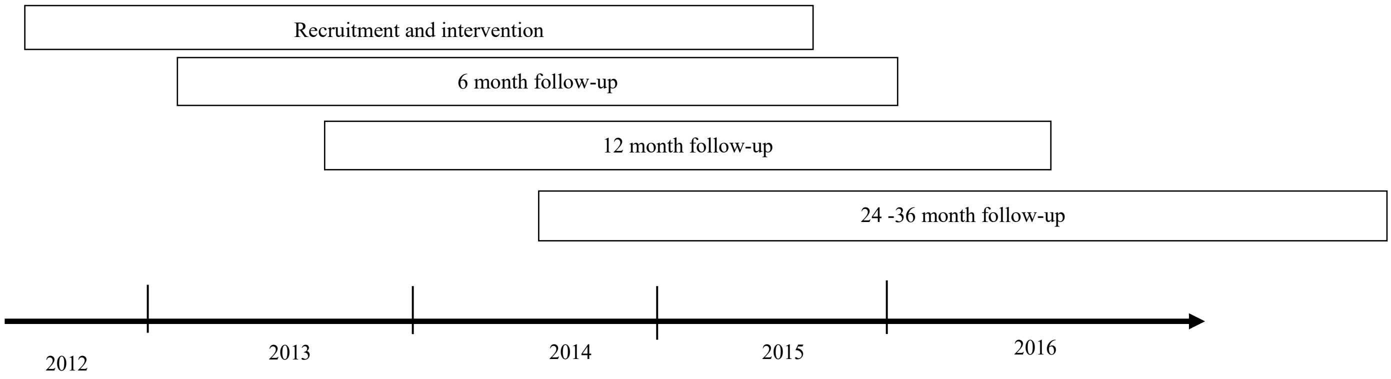

Significance

Societal and patient level
This research program has the potential to fill current knowledge gaps in the literature by providing evidence for the effect of brace treatment or physiotherapeutic intervention on scoliosis. If treatment is ineffective, the subsequent conclusion may be that the current school screening should be terminated, or at least modified.

Gender differences
In epidemiological studies, idiopathic scoliosis has been shown to be more prevalent in females. There are no reports on the importance of gender on the effectiveness of conservative interventions for idiopathic scoliosis or gender differences in factors moderating or mediating treatment. The impact of gender on the results will be specifically analysed in the publications.

Ethical Considerations

Today, patients with moderate scoliosis are offered treatment with a brace. The existing evidence for this treatment is poor and the effect not clear. It would be unethical not to perform a randomised controlled trial to study the efficacy of treatment.

Approval has recently been obtained by the regional ethics board in Stockholm (Dnr 2012/172-31/4) and the radiation committee at the Karolinska University Hospital (Dnr 2/12). No medical risks or obvious issues from a patient's integrity standpoint are associated with participation in the studies.

Implementation

It is expected that the publication of research results in peer reviewed scientific journals and presentation of results at national and international meetings and congresses will help with the wide implementation of results.

The principal investigators experience

Paul Gerdhem, associate professor and consultant in Orthopaedics, has extensive experience in handling large studies for longer time periods. His expertise involves more than 10 years of clinical management of scoliosis, and ongoing research including aetiological aspects of scoliosis. He will be principal investigator and also responsible for patient recruitment during his clinical work.

Allan Abbott, PhD, physiotherapist, has worked clinically with idiopathic scoliosis patients for over 10 years. Earlier research has investigated the influence of psychological factors showing them to be strong mediators explaining up to 50% of the disability and quality of life problems observed in chronic back pain patients. We have even observed that psychological factors predict the outcome of spinal surgery and that a rehabilitation approach focused on behavioural medicine and physical activity improves outcome in these patients [21-24]. We now shift focus of our research to investigate interventions to reduce the need for spinal correction surgery. Allan Abbott will be responsible for the postural intervention and the self-assessed physical activity part of the study.

Hans Möller, PhD and consultant in Orthopaedics, has extensive experience in performing clinical randomized controlled trials. He has 20 years experience in treatment of patients with idiopathic scoliosis and other spinal deformities. He will be responsible for patient recruitment during his clinical work.

References.

1. Weinstein, S.L., et al., *Health and function of patients with untreated idiopathic scoliosis: a 50-year natural history study.* JAMA, 2003. **289**(5): p. 559-67.
2. Anonymous, *Guidelines for school health care. National Board of Health and Welfare in Sweden. [In Swedish: Socialstyrelsens riktlinjer för skolhälsovården]. http://www.socialstyrelsen.se/Lists/Artikelkatalog/Attachments/10467/2004-130-2_20041302x.pdf.* 2011.
3. Pahys, J.M., et al., *Neurologic injury in the surgical treatment of idiopathic scoliosis: guidelines for assessment and management.* J Am Acad Orthop Surg, 2009. **17**(7): p. 426-34.
4. Li, X.F., et al., *Low bone mineral status in adolescent idiopathic scoliosis.* Eur Spine J, 2008. **17**(11): p. 1431-40.
5. McKay, H.A., et al., *Augmented trochanteric bone mineral density after modified physical education classes: a randomized school-based exercise intervention study in prepubescent and early pubescent children.* J Pediatr, 2000. **136**(2): p. 156-62.
6. Berg, U., ed. *Children and younger people. In: FYSS – Physical activity in the prevention treatment of disease. Ståhle A, Editor. Profession associations for physical activity and the Swedish National Institute of Public Health: Stockholm.* 2010.
7. Anonymous, *Skolbarns vanor inom fysisk aktivitet, tv-tittande och datoranvändning. Östersund: Statens Folkhälsoinstitut.* 2011.
8. Parsch, D., et al., *Sports activity of patients with idiopathic scoliosis at long-term follow-up.* Clin J Sport Med, 2002. **12**(2): p. 95-8.
9. Anonymous, *Nordic Council of Ministers. Nordic nutrition recommendations. Integrating nutrition and physical activity. NORD 2004:13. Copenhagen.* 2004.
10. WHO, *Global recommendations on physical activity for health. Geneva: World Health Organization [WHO]. .* 2010.
11. Kenanidis, E., et al., *Adolescent idiopathic scoliosis and exercising: is there truly a liaison?* Spine (Phila Pa 1976), 2008. **33**(20): p. 2160-5.
12. Weiss, H.R., et al., *Indications for conservative management of scoliosis (guidelines).* Scoliosis, 2006. **1**: p. 5.

13. Dolan, L.A. and S.L. Weinstein, *Surgical rates after observation and bracing for adolescent idiopathic scoliosis: an evidence-based review.* Spine (Phila Pa 1976), 2007. **32**(19 Suppl): p. S91-S100.
14. Fusco, C., et al., *Physical exercises in the treatment of adolescent idiopathic scoliosis: an updated systematic review.* Physiother Theory Pract, 2011. **27**(1): p. 80-114.
15. Maruyama, T., T.B. Grivas, and A. Kaspiris, *Effectiveness and outcomes of brace treatment: a systematic review.* Physiother Theory Pract, 2011. **27**(1): p. 26-42.
16. Bunge, E.M., J.D. Habbema, and H.J. de Koning, *A randomised controlled trial on the effectiveness of bracing patients with idiopathic scoliosis: failure to include patients and lessons to be learnt.* Eur Spine J, 2010. **19**(5): p. 747-53.
17. Danielsson, A.J., et al., *Health-related quality of life in patients with adolescent idiopathic scoliosis: a matched follow-up at least 20 years after treatment with brace or surgery.* Eur Spine J, 2001. **10**(4): p. 278-88.
18. Wessberg, P. and A. Nordwall, *Night-time Providence bracing compared to full time Boston bracing in adolescent idiopathic scoliosis. A prospective randomized study (Abstract). In: SICOT/SIROT/SOF; Aug 30-Sep 3; 2010 Aug 30-Sep 3; Gothenburg.* 2010.
19. Negrini, S., et al., *Braces for idiopathic scoliosis in adolescents.* Cochrane Database Syst Rev, 2010(1): p. CD006850.
20. Cobb, J., *Outline for the study of scoliosis. Instructional Course Lectures, The American Academy of Orthopaedic Surgeons (AAOS): Ann Arbor; p.261-275.* 1948.
21. Abbott, A.D., R. Hedlund, and R. Tyni-Lenne, *Patients' experience post-lumbar fusion regarding back problems, recovery and expectations in terms of the International Classification of Functioning, Disability and Health.* Disabil Rehabil, 2011. **33**(15-16): p. 1399-408.
22. Abbott, A.D., R. Tyni-Lenne, and R. Hedlund, *The influence of psychological factors on pre-operative levels of pain intensity, disability and health-related quality of life in lumbar spinal fusion surgery patients.* Physiotherapy, 2010. **96**(3): p. 213-21.
23. Abbott, A.D., R. Tyni-Lenne, and R. Hedlund, *Early rehabilitation targeting cognition, behavior, and motor function after lumbar fusion: a randomized controlled trial.* Spine (Phila Pa 1976), 2010. **35**(8): p. 848-57.
24. Abbott, A.D., R. Tyni-Lenne, and R. Hedlund, *Leg pain and psychological variables predict outcome 2-3 years after lumbar fusion surgery.* Eur Spine J, 2011. **20**(10): p. 1626-34.

SAP CONTRAIS

Draft : 2021.10.25

Analyses to be conducted BEFORE the primary outcome analysis.

- Create a variable (Treatment 1, 2, 3) to blind the statisticians.
- Inter-rater (ICC tests) for measurements of Cobb angle between AO and KJ (mean value) and measurements during the clinical evaluations.

PRIMARY OUTCOME

Statisticians: Per Näsman and Henrik Hedevik.

- Table with baseline characteristics
- Analysis of Variance with Dunnett´s correction mean Cobb angle of the major curve between night-time brace or scoliosis-specific exercise and control.
- Variables to be presented in a table: Age, gender, BMI, ATR, Risser, Distribution of the major curve, Cobb angle of the major curve.

ITT analysis

- Time the patients remained in the study in every group; Dunnett's t Tests for Duration.
- Comparison treatment 1 vs treatment 3 (Chi-Square test). Key variable: Progress Yes/No
- Comparison treatment 2 vs treatment 3 (Chi-Square test). Key variable: Progress Yes/No

Per-protocol analysis

- Time the patients remained in the study in every group; Dunnett's t Tests for Duration.
- Comparison treatment 1 vs treatment 3 (Chi-Square test). Key variable: Progress Yes/No
- Comparison treatment 2 vs treatment 3 (Chi-Square test). Key variable: Progress Yes/No

Kaplan-Meier survival analysis and log rank test

- probability of 6 degree or less Cobb progression over time for each group

Complier-average causal effect (CACE) analysis

- Dichotomous variables on patient adherence, motivation, capability
- Intention-to Treat and per-protocol CACE weighted analysis
    - Hazard Ratio with CIs
    - Adjustment for Age, Risser, Cobb major curve, Sex

Summary of changes from original statistical analysis plan (SAP).

- Inter-rater reliability (ICC) was conducted by one of the authors, AC, before the main analysis. This analysis was not stated in the published protocol.

- KM survival analysis was conducted to investigate on the probability of 6 degrees or less Cobb progression of the major curve and not on the probability of more than 6 degrees as stated in the published protocol.
