# Supplementary material for: Nighttime Bracing or Exercise in Moderate-Grade Adolescent Idiopathic Scoliosis: A Randomized Clinical Trial
Source: JAMA Netw Open. 2024 Jan 29;7(1):e2352492. doi: 10.1001/jamanetworkopen.2023.52492 (PMC10825714; doi:10.1001/jamanetworkopen.2023.52492)
Supplement: Supplement 2. — eTable 1. Causal Survival Analysis for Nighttime Bracing Compared With Self-Mediated Physical Activity eTable 2. Causal Survival Analysis for Scoliosis-Specific Exercise Compared With Self-Mediated Physical Activity [file jamanetwopen-e2352492-s002.pdf]

Supplemental Online Content

Charalampidis A, Diarbakerli, E, Dufvenberg M, et al; CONTRAIS Study Group. Effects of nighttime bracing or exercise in moderate-grade adolescent idiopathic scoliosis: a randomized clinical trial. *JAMA Netw Open*. 2024;7(1):e2352492. doi:10.1001/jamanetworkopen.2023.52492

eTable 1. Causal Survival Analysis for Nighttime Bracing Compared With Self-Mediated Physical Activity

eTable 2. Causal Survival Analysis for Scoliosis-Specific Exercise Compared With Self-Mediated Physical Activity

This supplemental material has been provided by the authors to give readers additional information about their work.

© 2024 Charalampidis A et al. *JAMA Network Open*.

eTable 1. Causal Survival Analysis for Nighttime Bracing Compared With Self-Mediated Physical Activity

	Nighttime brace	Physical activity	Pooled logistic regression	
Intention-to Treat analysis[1] N=90	Probability of 3-year survival* = 0.73	Probability of 3-year survival* = 0.48	Log Odds (95% CI)	-1.75 (-2.7 to -0.8)
			SE	0.47
			HR (95%)	0.17 (0.07 to 0.43)
			p-value	<0.001
Intention-to Treat CACE weighted analysis[2] N=90	Probability of 3-year survival* = 0.74	Probability of 3-year survival* = 0.47	Log Odds (95% CI)	-1.81 (-3.0 to -0.7)
			SE	0.60
			HR (95% CI)	0.16 (0.05 to 0.52)
			p-value	0.002
Per-Protocol (complete cases) CACE weighted analysis[2] N=80	Probability of 3-year survival* = 0.77	Probability of 3-year survival* = 0.44	Log Odds (95% CI)	-1.99 (-3.2 to -0.8)
			SE	0.59
			HR (95% CI)	0.14 (0.04 to 0.44)
			p-value	<0.001

* Survival was defined as skeletal maturity with 6 degrees or less of curve progression.

1 Adjusted for baseline covariates (Age, Risser, Cobb major curve, Sex).

2 Adjusted for baseline covariates (Age, Risser, Cobb major curve, Sex) and inverse probability weighted for post-randomization time-varying covariates (health care professional reported patient treatment adherence, patient reported treatment adherence, patient reported motivation to carry out treatment, patient reported capability to perform treatment).

SE = Standard error, HR= Hazard ratio, CACE= Complier Average Causal Effects, p<0.05 indicates statistical significance.

© 2024 Charalampidis A et al. *JAMA Network Open*.

eTable 2. Causal Survival Analysis for Scoliosis-Specific Exercise Compared With Self-Mediated Physical Activity

	**Scoliosis-specific exercise**	**Physical activity**	**Pooled logistic regression**	
Intention-to Treat analysis[1]<br>N=90	Probability of 3-year survival* = 0.45	Probability of 3-year survival* = 0.49	Log Odds (95% CI)<br>SE<br>HR (95%)<br>p-value	-0.10 (-0.9 to 0.7)<br>0.39<br>0.91 (0.43 to 1.93)<br>0.80
Intention-to Treat CACE weighted analysis[1]<br>N=90	Probability of 3-year survival* = 0.54	Probability of 3-year survival* = 0.49	Log Odds (95% CI)<br>SE<br>HR (95% CI)<br>p-value	-0.55 (-1.6 to 0.5)<br>0.53<br>0.58 (0.20 to 1.63)<br>0.30
Per-Protocol (complete cases) CACE weighted analysis[1]<br>N=80	Probability of 3-year survival* = 0.52	Probability of 3-year survival* = 0.47	Log Odds (95% CI)<br>SE<br>HR (95% CI)<br>p-value	-0.56 (-1.6 to 0.5)<br>0.52<br>0.57 (0.21 to 1.58)<br>0.28

* Survival was defined as skeletal maturity with 6 degrees or less of curve progression.

[1] Adjusted for baseline covariates (Age, Risser, Cobb major curve, Sex).

[2] Adjusted for baseline covariates (Age, Risser, Cobb major curve, Sex) and inverse probability weighted for post-randomization time-varying covariates (health care professional reported patient treatment adherence, patient reported treatment adherence, patient reported motivation to carry out treatment, patient reported capability to perform treatment).

SE = Standard error, HR= Hazard ratio, CACE= Complier Average Causal Effects, p<0.05 indicates statistical significance.

© 2024 Charalampidis A et al. *JAMA Network Open*.
