# Supplementary material for: Nighttime Bracing or Exercise in Moderate-Grade Adolescent Idiopathic Scoliosis: A Randomized Clinical Trial
Source: JAMA Netw Open. 2024 Jan 29;7(1):e2352492. doi: 10.1001/jamanetworkopen.2023.52492 (PMC10825714; doi:10.1001/jamanetworkopen.2023.52492)
Supplement: Supplement 3. — Nonauthor Collaborators. CONTRAIS Study Group members [file jamanetwopen-e2352492-s003.pdf]

Supplemental Online Content: Nonauthor Collaborators
*First name, last name, and suffix (if applicable) are required and will appear in PubMed.

*Group Name: CONTRAIS Study Group							
***First Name and Middle Initial(s)**	***Last Name**	***Suffix (eg, Jr, III)**	**Academic Degrees**	**Institution**	**Location (city, state/province, country)**	**Role or Contribution, eg, chair, principal investigator**	**Group (if more than 1 Group listed in the byline) and/or Subgroup (eg, Steering Committee)**
Birgitta	Öberg		RPT, PhD	Department of Health, Medicine and Caring Sciences, Unit of Physiotherapy, Linköping University	Linköping, Sweden	Patient recruitment, patient follow-up, data-analysis	
Hans	Tropp		MD, PhD	Department of Orthopaedics, Linköping University Hospital	Linköping, Sweden	Patient recruitment, patient follow-up, data-analysis	
Anna	Grauers		MD, PhD	Sundsvall and Harnosand County Hospital	Sundsvall, Sweden	Patient recruitment, patient follow-up	
Ylva	Bodén		MD	Department of Orthopaedics, Linköping University Hospital	Linköping, Sweden	Patient recruitment, patient follow-up	
Mats	Hoffsten		BSc	Team Olmed	Stockholm, Sweden	Senior Orthotist, responsible for brace introduction and adjustment.	
Per	Näsman		PhD	Center for Safety Research, KTH Royal Institute of Technology	Stockholm, Sweden	Statistical analysis	
Henrik	Hedevik		MSc	Department of Health, Medicine and Caring Sciences, Unit of Physiotherapy, Linköping University	Linköping, Sweden	Statistical analysis	
