# Supplementary material for: Nighttime Bracing or Exercise in Moderate-Grade Adolescent Idiopathic Scoliosis: A Randomized Clinical Trial
Source: JAMA Netw Open. 2024 Jan 29;7(1):e2352492. doi: 10.1001/jamanetworkopen.2023.52492 (PMC10825714; doi:10.1001/jamanetworkopen.2023.52492)
Supplement: Supplement 4. — Data Sharing Statement [file jamanetwopen-e2352492-s004.pdf]

Data Sharing Statement

Charalampidis. Nighttime Bracing or Exercise in Moderate-Grade Adolescent Idiopathic Scoliosis. *JAMA Netw Open*. Published January 29, 2024.
doi:10.1001/jamanetworkopen.2023.52492

Data
Data available: Yes
Data types: Deidentified participant data, Data dictionary
How to access data: Deidentified data will be available for sharing by the corresponding author upon reasonable request.
When available: With publication

Supporting Documents
Document types: Statistical/analytic code
How to access documents: Data will be available for sharing by the corresponding author upon reasonable request.
When available: With publication

Additional Information
Who can access the data: researchers whose proposed use of the data has been approved.
Types of analyses: Meta-analysis, systematic review
Mechanisms of data availability: After approval of a proposal, or with a signed data access agreement.
